# Antimicrobial susceptibility testing in predicting the presence of carbapenemase genes in Enterobacteriaceae in South Africa

**DOI:** 10.1186/s12879-016-1858-7

**Published:** 2016-10-04

**Authors:** Ashika Singh-Moodley, Olga Perovic

**Affiliations:** 1 Division of the National Health Laboratory Service, National Institute for Communicable Diseases, 1 Modderfontein Road, Sandringham, 2131 Johannesburg, South Africa; 2 University of Witwatersrand, South Africa, Private Bag 3, Wits 2050, Johannesburg, South Africa

**Keywords:** Carbapenemase-producing Enterobacteriaceae, Antimicrobial susceptibility testing, Resistance genes, Carbapenemases

## Abstract

**Background:**

Carbapenem-resistant Enterobacteriaceae (CRE) is a concern in South Africa and worldwide. It is therefore important that these organisms be accurately identified for infection prevention control purposes.

**Method:**

In this study 1193 suspected CREs from 46 laboratories from seven provinces in South Africa were assessed to confirm the prevalence of carbapenemase genes from our referral diagnostic isolates for the period 2012 to 2015. We compared the antimicrobial susceptibility testing method used in the reference laboratory to the polymerase chain reaction (PCR) which is used as the gold standard. Organism identification and antimicrobial susceptibility testing were performed using automated systems and DNA was extracted using a crude boiling method. The presence of carbapenemase-producing genes (*bla*_NDM,_ *bla*_KPC,_ *bla*_OXA-48_&variants_,_ *bla*_GES,_ *bla*_IMP_ and *bla*_VIM_) was screened for using a multiplex real-time PCR.

**Results:**

Sixty-eight percent (*n* = 812) of the isolates harboured a carbapenemase-producing gene; the three most common genes included: *bla*_NDM,_ *bla*_OXA-48_&variants and *bla*_VIM_. Majority of the carbapenemase producing Enterobacteriaceae (CPE) isolates were *Klebsiella* species (71 %). The Microscan® Walkaway system used for the screening of carbapenemase production was 98 % sensitive with a minimal inhibitory concentration (MIC) breakpoint of less than 0.5 as susceptible for ertapenem and a low specificity (13 %).

**Conclusion:**

From this study we can conclude that carbapenemase-producing Enterobacteriaceae is increasing in South Africa and the use of phenotypic methods for detection of CPEs showed good sensitivity but lacked specificity.

## Background

Multidrug resistant organisms are a major public health concern [1, 2]. There is an increase in the global detection of antibiotic resistant Enterobacteriaceae strains with antibiotic resistance observed to beta-lactams, fluoroquinolones, aminoglycosides and polymixins [3]. Beta-lactam resistance mechanisms include outer membrane permeability changes, efflux pumps and enzymes that hydrolyse the antibiotic for example, the carbapenemases, including: the metallo-beta-lactamases (MBLs), NDM, IMP and VIM; the oxacillinases, OXA-48 and non-metallo-enzymes, *Klebsiella pneumoniae* carbapenemases (KPC) [3]. The emergence of resistance to the broad-spectrum carbapenem group of antibiotics is particularly worrying as it limits treatment options and necessitates the need for the use of antibiotics like tigecycline and colistin which contribute to toxicity [4–6]. Carbapenemase-producing Enterobacteriaceae (CPE) are carbapenem-resistant Enterobacteriaceae (CRE) that are resistant to carbapenems due to carbapenemase production [7]. Carbapenemases are beta-lactamases [8] that hydrolyse almost all beta-lactams including the carbapenems [3, 9]. Carbapenemases were initially chromosomally-mediated in a few specific species, however majority are now plasmid-mediated, or both chromosomally- and plasmid-mediated [10, 11] resulting in horizontal transmission among various bacterial species and genera. The ability of these mobile genetic elements (plasmids) to acquire, harbour and disseminate multiple resistance genes contributes to the successful and aggressive spread of resistant organisms [12]. The presence of these genes has been documented worldwide such as in Asia, Europe, Australia and Africa including South Africa [13–32]. In South Africa the first case of NDM in *Enterobacter cloacae* was reported in the Gauteng province in 2011 and then almost a year later in KwaZulu-Natal. *Klebsiella pneumoniae* isolates harbouring KPC, OXA-48 and OXA-181were reported in Gauteng 2011. An outbreak of OXA-181 in *Klebsiella pneumoniae* was documented in the Western Cape in 2012 and the presence of OXA-48, VIM and IMP in *Enterobacter cloacae* isolates isolated from patients in the Eastern Cape has also been reported [27–32]. The need to prevent the spread of these organisms is important, however, there are some obstacles. Many clinical laboratories do not routinely test for CRE resistance-mechanisms. They are therefore not able to differentiate between non-susceptible CREs (which may be as a result of carbapenemases or *via* combinations of ampicillin class C (AmpC) enzymes, extended-spectrum beta-lactamases (ESBL) and membrane impermeability), and CPEs (which are as a result of carbapenemase production exclusively) [33]. Furthermore, the Clinical and Laboratory Standards Institute (CLSI) does not recommend molecular resistance-mechanism testing for clinical purposes [34].

In this study, we evaluated1193 suspected CREs to estimate the prevalence of CPEs in our sample population in South Africa. This was not a surveillance study but nonetheless provided us with baseline information regarding the extent of CPEs in the country and the organisms producing them. We also evaluated one of the phenotypic methods routinely used for antimicrobial susceptibility testing (AST) to determine its efficacy and accuracy and report on its ability to screen for CREs based on confirmed CPE genes received at the reference laboratory.

## Methods

The national Antimicrobial Resistance Laboratory (AMRL) for the public healthcare sector, which majority of the South African population have access to, have been testing referred suspected CPE isolates for the presence of selected carbapenemases. Clinically significant phenotypically identified carbapenem non-susceptible Enterobacteriaceae isolates were voluntarily submitted from 46 laboratories to the AMRL at the Centre for Opportunistic, Tropical and Hospital Infections (COTHI), National Institute for Communicable Diseases (NICD) for confirmation of carbapenemase-producing genes as part of a routine diagnostic programme. Isolates received for the period 2012–2015 were analysed; it is important to note that each isolate was analysed individually (for 179 patients, isolates from multiple sites and some repeats were submitted). When the isolate was received at the AMRL, organism identification was performed using automated systems (VITEK^®^ II (bioMèrieux, France) and/or the Microflex MALDI-ToF (Bruker Daltonik, GmbH) and antimicrobial susceptibility testing (AST) was carried out on the automated MicroScan^®^ Walkaway system (Siemens, USA) using the Gram-negative MIC Panel Type 44 (ertapenem 0.5–1μg/mL; doripenem 1–4μg/mL; imipenem 1–8 μg/mL; meropenem 1–8 μg/mL). The Sensititre instrument (Trek Diagnostic Systems, UK) was used to clarify and confirm questionable Microscan results (i.e. an isolate was phenotypically susceptible to the carbapenems although a carbapenemase was present). For this procedure, microtitre trays with two-fold dilutions of antibiotics were inoculated with a standardised inoculum of the bacteria and incubated under standardised conditions. The minimal inhibitory concentration (MIC) was recorded the next day as the lowest concentration of antimicrobial agent with no visible growth. The interpretation of susceptibility was done according to the Clinical and Laboratory Standards Institute (CLSI) guidelines [34]. For genotypic testing, DNA was extracted using a crude boiling method at 95°C for 25 minutes for cell lysis. The supernatant was harvested and screened for *bla*_NDM_, *bla*_KPC_, *bla*_OXA-48_ and its variants (OXA-162, 163, 244 245, 247, 181, 204, 232), *bla*_GES_ (GES-1-9, 11)_,_ *bla*_IMP_ (IMP-9, 16, 18, 22, 25) and *bla*_VIM_ (VIM-1-36) using a multiplex real-time polymerase chain reaction (PCR) (LightCycler 480 II, Roche Applied Science, LightCycler 480 Probes Master kit and the individual LightMix Modular kits (Roche Diagnostics, IN, USA).

Statistical analyses were performed using Graph Pad Prism 5.

## Results

In this study, in the period 2012–2015, we received 1193 suspected CREs to confirm CPE genes. We also evaluated the phenotypic method used for antimicrobial susceptibility testing and reporting of CREs.

From a total of 1193 isolates, 68 % (*n* = 812) harboured at least one or a combination of CPE genes. Of these, 469 (57 %) were *bla*_NDM_-positive, 219 (26 %) were *bla*_OXA-48_-positive (including variants), 89 (10 %) were *bla*_VIM_-positive, 35 (4 %) were *bla*_IMP_-positive, 18 (2 %) were *bla*_GES_-positive and 11 (1 %) were *bla*_KPC_-positive. Combinations consisted of *bla*_NDM_- and *bla*_OXA-48_-positive (*n* = 8), *bla*_NDM_- and *bla*_IMP_-positive (*n* = 4), *bla*_NDM_- and *bla*_VIM_-positive (*n* = 3), *bla*_NDM_- and *bla*_KPC_-positive, *bla*_KPC_- and *bla*_VIM_-positive, *bla*_IMP_- and *bla*_OXA-48_-positive, *bla*_VIM_- and *bla*_OXA-48_-positive (*n* = 2 each) and *bla*_NDM_- and *bla*_GES_-positive and *bla*_GES_- and *bla*_OXA-48_-positive (*n* = 1 each).

**Fig. 1:**
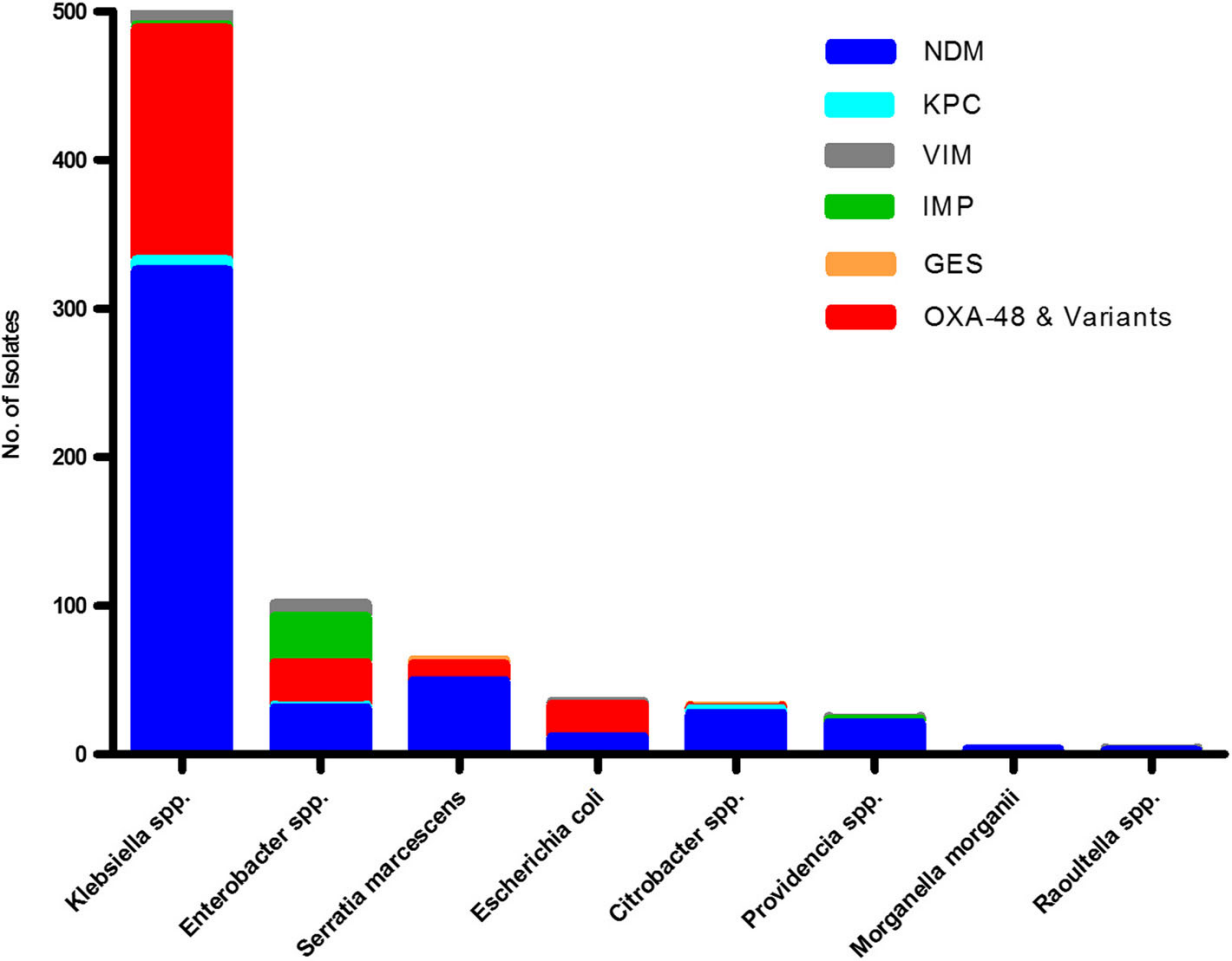
Organism and genotype information in carbapenemase producing isolates

Majority of the carbapenemase producing isolates were *Klebsiella* spp. (71 %, *n* = 580) followed by *Enterobacter* spp. (12 %, *n* = 101), *Serratia marcescens* (7.8 %, *n* = 63), *Escherichia coli* (4 %, *n* = 35), *Citrobacter* spp. (3.9 %, *n* = 32), *Providencia* spp. (2.9 %, *n* = 24) and *Morganella morganii* and *Raoultella* spp. (0.4 %, *n* = 3, each). The predominating genotype in most organism species was *bla*_NDM_ with the exception of *Enterobacter* spp. which also included *bla*_IMP_. In *E. coli* the predominating genotype was *bla*_OXA-48_ and its variants which were also commonly found in all other organisms except in *Providencia* spp., *Morganella morganii* and *Raoultella* spp. (Fig. 1).

**Fig. 2:**
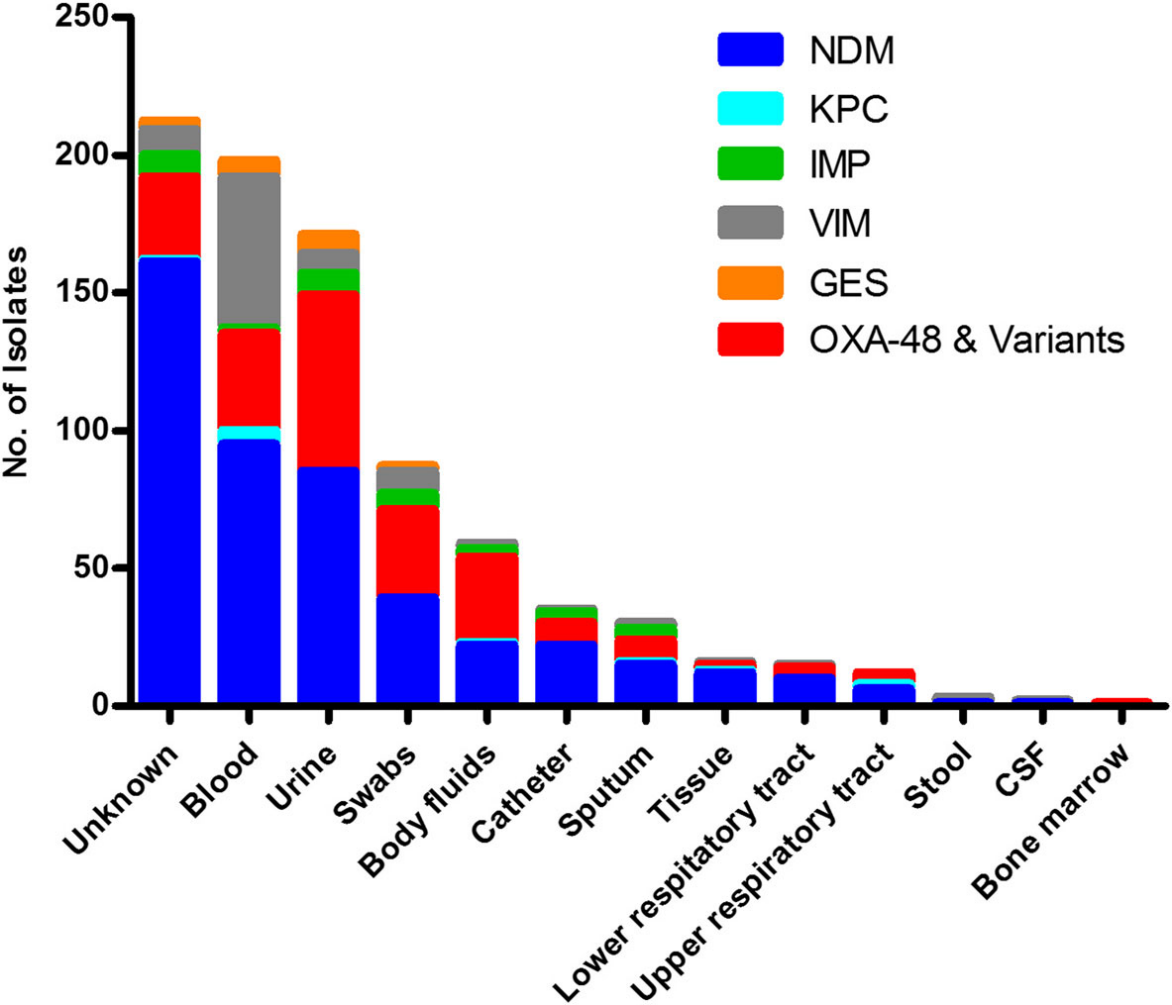
Specimen type and genotype information in carbapenemase producing isolates. *Citrobacter* spp.: NDM, *n* = 27; KPC, *n* = 3; OXA-48, *n* = 1; GES, *n* = 1. Enterobacter spp.: NDM, *n* = 31; KPC, *n* = 1; OXA-48, *n* = 29; IMP, *n* = 31; VIM, *n* = 9. E.coli: NDM, *n* = 11; OXA-48, *n* = 22, VIM, *n* = 2. Klebsiella spp.: NDM, *n* = 325; KPC, *n* = 7; OXA-48, *n* = 156; IMP, *n* = 2; VIM, *n* = 76; GES, *n* = 14. Morganella morganii: NDM, *n* = 3. Providencia spp.: NDM, *n* = 21, IMP, *n* = 2; VIM, *n* = 1. Raoultella spp.: NDM, *n* = 2. Serratia marcescens: NDM, *n* = 49; OXA-48, *n* = 11; GES, *n* = 3

Specimen types in the carbapenemase producing isolates were blood (32 %, *n* = 257), urine (21 %, *n* = 171), swabs (11 %, *n* = 87), body fluids (7 %, *n* = 59), catheter tip (4 %, *n* = 35), sputum (3 %, *n* = 30) and smaller percentages ranging from 0.1 % (*n* = 1) to 1.9 % (*n* = 16) for bone marrow, cerebrospinal fluid, stool, upper respiratory tract, lower respiratory tract and tissue specimens. Up to 26 % (*n* = 212) of the isolates specimen types were unknown or the data were not available (Fig. 2). There was no apparent correlation/relationship between specimen type and organism or specimen type and genotype.

**Fig. 3:**
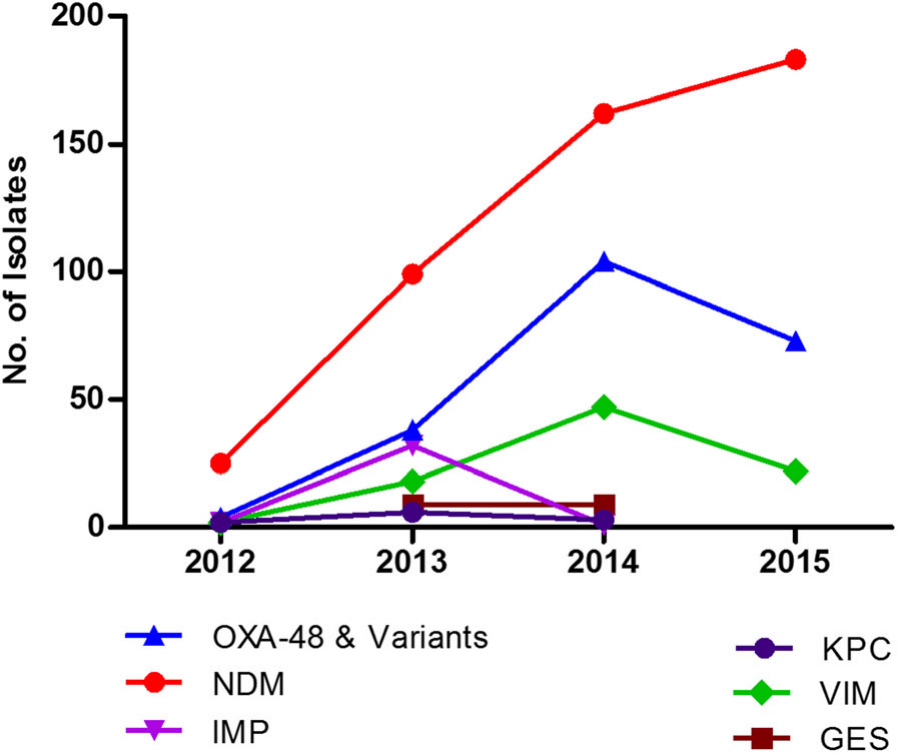
Genotype information in carbapenemase producing isolates over the 3 year period. 2012 – NDM *n* = 25, 71 %; KPC *n* = 2, 6 %; OXA-48 *n* = 4, 11 %; IMP *n* = 2, 6 %; VIM *n* = 2, 6 %; GES *n* = 0. 2013 – NDM *n* = 99, 49 %; KPC *n* = 6, 3 %; OXA-48 *n* = 38, 19 %; IMP *n* = 32, 16 %; VIM *n* = 18, 9 %; GES *n* = 9, 4 %. 2014 – NDM *n* = 162, 50 %; KPC *n* = 3, 1 %; OXA-48 *n* = 104, 32 %;IMP *n* = 1, 0.3 %;VIM *n* = 47, 14 %;GES *n* = 9, 3 %. 2015 – NDM *n* = 183, 66 %; KPC *n* = 0; OXA-48 *n* = 73, 26 %; IMP *n* = 0; VIM *n* = 22, 8 %; GES *n* = 0

*Bla*_NDM_ predominated in the years 2012–2015 (71 %, *n* = 25; 49 %, *n* = 99; 50 %, *n* = 162 and 66 %, *n* = 183 respectively) followed by *bla*_OXA-48_ and its variants (11 %, *n* = 4; 19 %, *n* = 38; 32 %, *n* = 104 and 26 %, *n* = 73 respectively). In 2013, 16 % (*n* = 32) were *bla*_IMP_-positive whereas in 2014 and 2015 there were higher numbers of *bla*_VIM_-positive isolates (14 %, *n* = 47 and 8 %, *n* = 22 respectively) (Fig. 3). Statistical analysis using the chi-squared test revealed that the change in resistance mechanisms from 2012 to 2015 was significant (*p* < 0.0001) i.e. there was a significant increase in the number of genotypes detected from 2012 compared to 2015.

**Fig. 4:**
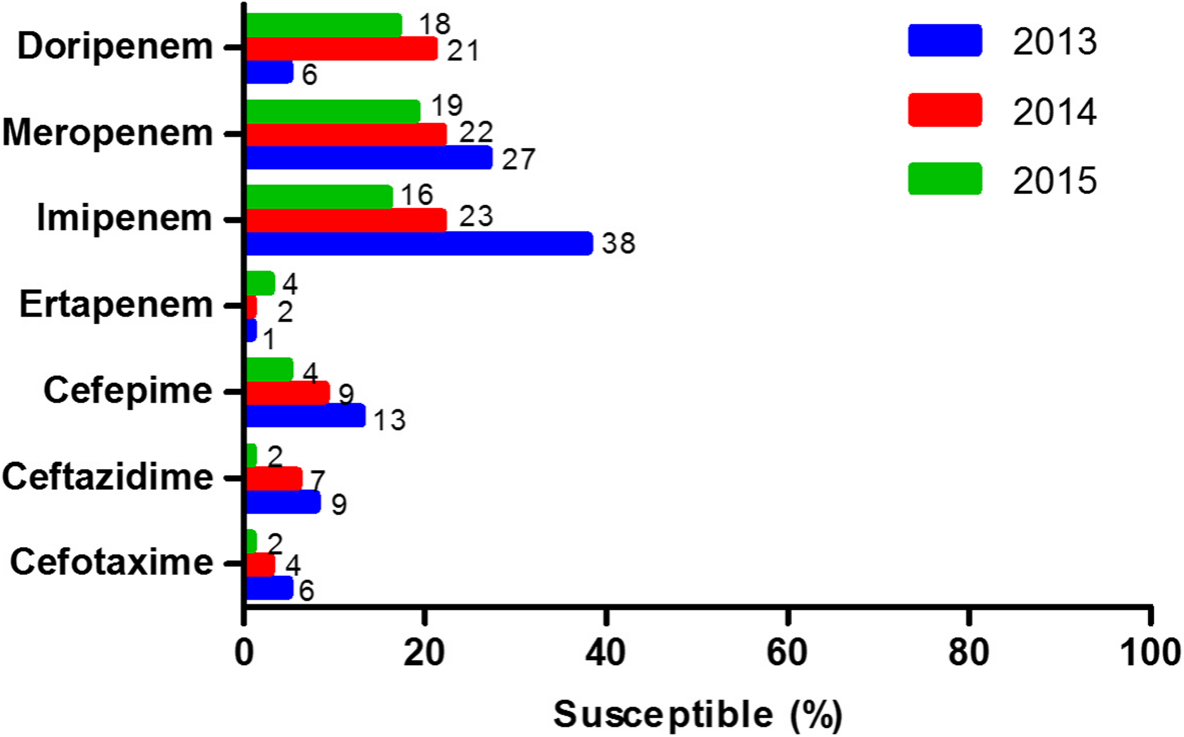
Percentage susceptibility to carbapenems and cephalosporins in carbapenemase producing isolates, 2013-2015. Data for 2012 was limited and was excluded from this analysis

Antimicrobial susceptibility testing profiles of these carbapenemase producing isolates were analysed. Ertapenem proved to be a reliable indicator of carbapenemase production. Ertapenem non-susceptibility was observed in 98 % (*n* = 794) of the carbapenemase producing isolates indicating that the phenotypic method used was fairly sensitive. Eighteen (2 %) isolates produced an ertapenem-susceptible result although a CPE gene was present (*bla*_VIM,_ *n* = 10; *bla*_NDM,_ *n* = 4; *bla*_VIM,_ and *bla*_OXA-48_-like_,_ *n* = 1; *bla*_IMP,_ *n* = 1; *bla*_GES,_ *n* = 1 and *bla*_OXA-48_ and variants_,_ *n* = 1). Three of these isolates were fully susceptible to the third and fourth generation cephalosporins (cefotaxime, ceftazidime and cefepime) as well as to the remaining carbapenems (imipenem, meropenem and doripenem). These isolates contained the following genes: *bla*_NDM_ (*n* = 2) and *bla*_OXA-48_ and variants (*n* = 1). Interestingly, 12 of these ertapenem-susceptible isolates showed non-susceptibility to imipenem; two of these were also non-susceptible to meropenem. Majority (92 %, *n* = 11) of these were *bla*_VIM_-positive with one isolate also positive for *bla*_OXA-48_ and its variants. The remaining two isolates contained *bla*_NDM_ (Table 1)_._ Of the carbapenem non-susceptible carbapenemase producing isolates, one isolate was susceptible to cefotaxime, 12 were susceptible to ceftazidime and 23 were susceptible to cefepime. The carbapenemase types are seen in Table 2. The percentage susceptibility for the cephalosporins and carbapenems is presented in Fig. 4. The susceptibility rates for cefotaxime, ceftazidime and cefepime decreased over the three year period. There was a slight increase in the percentage susceptibility for ertapenem over the three year period: 1 % (*n* = 2) in 2013, 1.6 % (*n* = 5) in 2014 and 3.72 % (*n* = 10) in 2015), however overall the percentage susceptibility was low.

**Table 1:** Minimal inhibitory concentrations (MIC) of ertapenem susceptible carbapenemase producing isolates

Sample ID	ORGANISM	NDM	KPC	OXA-48 & variants	IMP	VIM	GES	Cefepime MIC	Cefotaxime MIC	Ceftazidime MIC	Ertapenem MIC	Imipenem MIC	Meropenem MIC	Doripenem MIC
ML0148.2	*Enterobacter cloacae*	-	-	-	+	-	-	>16	>32	>16	<=0.5	<=2	<=1	Not tested
ML0191	*Klebsiella pneumoniae*	-	-	-	-	-	+	8	>32	>16	<=0.5	<=2	<=1	Not tested
ML0440	*Klebsiella pneumoniae*	-	-	-	-	+	-	16	>32	>16	<=0.5	4	2	Not tested
ML0446	*Klebsiella pneumoniae*	-	-	-	-	+	-	<=1	<=1	<=1	<=0.5	<=2	<=1	Not tested
ML0848	*Klebsiella pneumoniae*	-	-	-	-	+	-	>16	>32	>16	<=0.5	4	<=1	<=1
ML0917	*Providencia rettgeri*	+	-	-	-	-	-	<=1	<=1	<=1	<=0.5	<=1	<=1	<=1
ML0926	*Escherichia coli*	+	-	-	-	-	-	<=1	<=1	<=1	<=0.5	<=1	<=1	<=1
ML0930	*Klebsiella pneumoniae*	-	-	-	-	+	-	>16	>32	>16	<=0.5	4	<=1	<=1
ML1002	*Providencia rettgeri*	+	-	-	-	-	-	8	32	>16	<=0.5	>8	2	2
ML1007	*Klebsiella pneumoniae*	-	-	-	-	+	-	>16	>32	>16	<=0.5	4	<=1	2
ML1161	*Klebsiella pneumoniae*	-	-	+	-	-	-	>16	>32	>16	<=0.5	<=2	<=1	Not tested
ML1187	*Escherichia coli*	-	-	+	-	+	-	2	8	16	<=0.5	4	<=1	<=1
ML1189	*Klebsiella pneumoniae*	-	-	-	-	+	-	>16	>32	>16	<=0.5	2	<=1	<=1
ML1219	*Klebsiella pneumoniae*	-	-	-	-	+	-	16	>32	>16	<=0.5	2	<=1	<=1
ML1279	*Klebsiella pneumoniae*	-	-	-	-	+	-	>16	>32	>16	<=0.5	2	<=1	<=1
ML1296	*Klebsiella pneumoniae*	-	-	-	-	+	-	>16	>32	>16	<=0.5	4	<=1	<=1
ML1340	*Morganella morganii*	+	-	-	-	-	-	8	>32	>16	<=0.5	8	<=1	2
ML1354	*Klebsiella pneumoniae*	-	-	-	-	+	-	>16	>32	>16	<=0.5	4	<=1	2

CLSI breakpoints: Cefepime: <=2 – Susceptible, 4-8 – Susceptible-Dose Dependant, > = 16 – Resistant; Cefotaxime: <=1 - Susceptible, 2 – Intermediate, > = 4 – Resistant; Ceftazidime: <=4 - - Susceptible, 8 – Intermediate, > = 16 – Resistant; Ertapenem: <=0.5- Susceptible, 1 – Intermediate, > = 2 – Resistant; Imipenem/Meropenem/Doripenem: <=1 – Susceptible, 2 – Intermediate, > = 4 Resistant [34]

**Table 2:** Minimal Inhibitory Concentrations (MIC) of carbapenem non-susceptible, cephalosporin susceptible carbapenemase producing isolates

Sample ID	Organism	NDM	KPC	OXA-48& variants	IMP	VIM	GES	Cefepime MIC	Cefotaxime MIC	Ceftazidime MIC	Ertapenem MIC	Imipenem MIC	Meropenem MIC	Doripenem MIC
ML0056	*Klebsiella oxytoca*	-	-	+	-	-	-	16	8	<=1	>4	>8	>8	Not tested
ML0273	*Klebsiella pneumoniae*	-	-	+	-	-	-	2	4	<=1	>1	>8	>8	>4
ML0344	*Providencia rettgeri*	+	-	-	+	-	-	8	32	>16	4	>8	4	Not tested
ML0432	*Serratia marcescens*	-	-	-	-	-	+	<=1	4	2	>4	>8	>8	Not tested
ML0484	*Klebsiella pneumoniae*	-	-	+	-	-	-	<=1	2	<=1	>1	>8	4	4
ML0513	*Klebsiella pneumoniae*	-	-	+	-	-	-	<=1	4	<=1	>1	4	8	4
ML0514	*Klebsiella pneumoniae*	-	-	+	-	-	-	<=1	2	<=1	>1	2	4	4
ML0515	*Klebsiella pneumoniae*	-	-	+	-	-	-	<=1	2	<=1	>1	4	>8	>4
ML0517	*Klebsiella pneumoniae*	-	-	+	+	-	-	<=1	2	<=1	>1	4	4	4
ML0634	*Serratia marcescens*	-	-	+	-	-	-	8	>32	16	>1	>8	>8	>4
ML0794	*Klebsiella pneumoniae*	-	-	+	-	-	-	<=1	<=1	<=1	>1	2	2	<=1
ML0825	*Serratia marcescens*	-	-	+	-	-	-	8	>32	16	>1	>8	>8	>4
ML0841	*Serratia marcescens*	-	-	-	-	-	+	8	32	8	>1	>8	>8	>4
ML0878	*Providencia rettgeri*	+	-	-	-	-	-	8	32	>16	>1	>8	>8	>4
ML0883	*Serratia marcescens*	-	-	+	-	-	-	8	>32	16	>1	8	>8	>4
ML0886	*Serratia marcescens*	-	-	+	-	-	-	8	>32	>16	>1	>8	>8	>4
ML0905	*Serratia marcescens*	-	-	+	-	-	-	8	4	2	>1	8	>8	>4
ML0910	*Enterobacter gergoviae*	-	-	+	-	-	-	4	4	<=1	>1	2	>8	>4
ML0918	*Providencia rettgeri*	+	-	-	-	-	-	8	32	>16	>1	>8	8	>4
ML0976	*Escherichia coli*	-	-	+	-	-	-	<=1	2	<=1	>1	2	4	4
ML0999	*Providencia rettgeri*	+	-	-	-	-	-	8	32	>16	>1	>8	>8	>4
ML1167	*Serratia marcescens*	-	-	+	-	-	-	8	>32	16	>1	>8	>8	>4
ML1176	*Providencia rettgeri*	+	-	-	-	-	-	8	16	>16	1	>8	8	>4
ML1411	*Klebsiella pneumoniae*	-	-	+	-	-	-	8	>32	8	>1	8	2	4

CLSI breakpoints: Cefepime: <=2 – Susceptible, 4-8 – Susceptible-Dose Dependant, > = 16 – Resistant; Cefotaxime: <=1 - Susceptible, 2 – Intermediate, > = 4 – Resistant; Ceftazidime: <=4 - - Susceptible, 8 – Intermediate, > = 16 – Resistant; Ertapenem: <=0.5- Susceptible, 1 – Intermediate, > = 2 – Resistant; Imipenem/Meropenem/Doripenem: <=1 – Susceptible, 2 – Intermediate, > = 4 Resistant [34]

Imipenem and meropenem were fairly good indicators of carbapenemase production with non-susceptibility observed in 77 % (*n* = 643) and 79 % (*n* = 661) of the carbapenemase producing isolates, respectively. A decrease in the percentage susceptibility was also seen for imipenem, meropenem and doripenem over time. It should be noted that the instrument panel did not initially consist of doripenem and it was therefore not tested for, accounting for the low percentage obtained in 2013.

Of the 381 non-CPE isolates 333 (87 %) were phenotypically non-susceptible to ertapenem; 88 (23 %) were non-susceptible to imipenem and 155 (41 %) were non-susceptible to meropenem indicating that the phenotypic method used lacks specificity particularly in the case of ertapenem. Majority of the non-CPE isolates were *Klebsiella* spp. (49 %, *n* = 185) followed by *Enterobacter* spp. (40 %, *n* = 154), *Escherichia coli* (6 %, *n* = 23), *Morganella morganii* (2.7 %, *n* = 10), *Serratia marcescens* (1.5 %, *n* = 6), *Proteus mirabilis.* (0.5 %, *n* = 2) and *Citrobacter freundii*. (0.3 %, *n* = 1). Specimen types varied: urine (27 %, *n* = 102), blood (20 %, *n* = 77), swabs (16 %, *n* = 60), body fluids (7 %, *n* = 28), catheter tip (4 %, *n* = 16), sputum (3.7 %, *n* = 14) and smaller percentages ranging from 0.3 % (*n* = 1) to 1.6 % (*n* = 6) for cerebrospinal fluid, upper respiratory tract, lower respiratory tract and tissue specimens. Up to 18 % (*n* = 70) of the isolates specimen types were unknown or the data were not available.

The overall antimicrobial susceptibility and PCR (gold standard) results were compared to determine the sensitivity, specificity, positive predictive value and negative predictive value of the phenotypic method routinely used (i.e. the MicroScan^®^ Walkaway system). The sensitivities, specificities, positive and negative predicted values for ertapenem, imipenem and meropenem can be seen in Table 3. The use of doripenem as a denominator was omitted as it was not included in the AST panel for all isolates tested.

**Table 3:** Sensitivity, specificity, positive and negative predictive values of the microscan walkaway system

Antibiotic	Sensitivity (%)	Specificity (%)	Positive predictive value (%)	Negative predictive value (%)
Ertapenem	98	13	70	73
Imipenem	76	77	88	60
Meropenem	78	59	80	56

## Discussion

This study is a snapshot of the distribution of CPEs over a period of three years and does not represent the current burden of CPEs in South Africa. This study elucidates the current prevalent circulating CPE types. We observed that a high percentage (68 %) of the isolates harboured at least one or a combination of carbapenemase-producing genes. It should be noted that although genotypic methods are remarkably reliable, there are some potential limitations. False-negative results may be obtained as genes may not be detected due to the presence of genetic variants and/or rare carbapenemase types that were not included in the screening e.g. SPM, SIM, IMI etc. This could suggest that variants and other carbapenemases may potentially have been missed suggesting that the actual prevalence of CPEs in our study population could be higher. This is worrisome as the rate of CPEs in South Africa is increasing as indicated by the number of studies reporting occurrences and outbreaks [27–32].

Majority of the CPE isolates (those harbouring carbapenemases) were *Klebsiella* spp*.* (71 %, *n* = 580) followed by *Enterobacter* spp. (12 %, *n* = 101), *Escherichia coli* (4 %, *n* = 35) and *Citrobacter* spp. (3.9 %, *n* = 32) were also present to a smaller extent. Interestingly, we observed that 10.7 % (*n* = 90) of the carbapenemase producing isolates belonged to one of the following organisms: *Serratia marcescens* (7.8 %, *n* = 63), *Providencia* spp. (2.9 %, *n* = 24) and *Morganella morganii* (0.4 %, *n* = 3). Carbapenem resistance is particularly worrying in organisms such as these as well as *Proteus* spp. as these organisms are intrinsically resistant to colistin and if carbapenem cannot be used for treatment, the management of patients infected with these organisms is all the more challenging. Overall, in addition to the carbapenems other antibiotics were analysed for all carbapenemase producing isolates; with the exception of a few (amikacin, tigecycline and colistin) which showed high susceptibility, majority of the isolates showed varying degrees of resistance to the other antibiotics (ciprofloxacin, gentamicin, trimethoprim/sulfamethoxazole, pipercillin/tazobactam, ampicillin/sulbactam, aztroenam, cefotaxime, ceftazidime, cefepime and cefpodoxime). Resistance to these antibiotics may also be due to the presence of AmpCs and/or ESBLs. Screening for these would provide useful information for management purposes, however this was not done.

The most predominant genotype in the carbapenemase producing Enterobacteriaceae group was *bla*_NDM_ (56 %, *n* = 469) followed by *bla*_OXA-48_ and its variants (26 %, *n* = 219). *Bla*_VIM,_ *bla*_IMP,_ *bla*_GES_ and *bla*_KPC_ were also present but on a smaller scale (11 %, *n* = 89; 4 %, *n* = 35; 2 %, *n* = 18 and 1 %, *n* = 11 respectively). This is consistent with previous findings in South Africa where these genotypes have been confirmed in both the public and private sector hospitals in various provinces [27, 28, 30–32, 35]. There was a marked increase in *bla*_NDM_ and *bla*_OXA-48_ and its variants from 2012 to 2015. This is a matter of concern as it may indicate a possible emergence of these genotypes which have the potential to become endemic. The significant increase in the number of genotypes detected from 2012 compared to 2015 further highlights this.

Phenotypically, ertapenem non-susceptibility is the most sensitive indicator of carbapenemase production [34]. This was observed in our study with ertapenem non-susceptibility observed in 98 % of the carbapenemase producing isolates. Of these isolates approximately 3 % (*n* = 24) were non-susceptible to ertapenem but susceptible to the cephalosporins. According to the Clinical and Laboratory Standards Institute, isolates displaying non-susceptibility to ertapenem usually also test resistant to one or more cephalosporin subclass III agents (e.g. cefoperazone, cefotaxime, ceftazidime and ceftriaxone), however some isolates producing carbapenemases still continue to test susceptible to these cephalosporins [34], a possible explanation for our outcomes. In summary, isolates displaying ertapenem-non-susceptibility and cephalosporin-susceptibility may often be OXA-48 producers without harbouring an ESBL, which could possibly be the case here. Three isolates were fully susceptible to the third and fourth generation cephalosporins as well as to imipenem, meropenem and doripenem. This is interesting because except for CREs that are OXA-48-like producers, most Enterobacteriaceae that produce carbapenemases are generally resistant to more than one antibiotic and resistant to third generation cephalosporins [33]. One of these isolates was an OXA-48 producer but the other two have shown to produce NDM, suggesting that some organisms do not conform to the traditional behaviour and patient management could perhaps be on an individual basis depending on AST profiles and resistance mechanisms. It should be noted that although the phenotypic result was confirmed with two methodologies (Microscan and Sensititre), the molecular result was only indicative of the multiplex real-time PCR used and alternative primers were not employed. Moreover, sequencing of *bla*_NDM_ was not performed which would further provide subtype information. Non-traditional AST profiles were also seen in other carbapenemase producing isolates which were carbapenem non-susceptible but susceptible to cefotaxime, ceftazidime or cefepime (Table 2). Majority of these were OXA-48 producers. Furthermore, it was observed that a small percentage (1.5 %, *n* = 12) of ertapenem-susceptible isolates showed non-susceptibility to imipenem with a further subset (0.3 %, *n* = 2) being non-susceptible to meropenem as well. Majority of these were VIM-producers suggesting that low MICs to ertapenem can be typical of certain VIM types, as VIM-producers have variable kinetics for the different carbapenems. Hence there is heterogeneity in expression within the same clone and amongst different species [36, 37] and may in some instances result in some VIM-types not being detected phenotypically. Two percent of the carbapenemase producing isolates displayed fully carbapenem susceptible profiles. Previous studies have shown that the MIC breakpoints for carbapenems in particular, imipenem and meropenem were not elevated and in the susceptibility range of <1 underscoring the difficulties in identifying metallo-β-carbapenemase- producing organisms primarily based on MIC results [16, 32].

While ertapenem has shown to be a reliable indicator of resistance in the carbapenemase producing Enterobacteriaceae group, phenotypic methods may not be ideal as observed in the non-CPE group as indicated by the high percentage (87 %) of non-CPE ertapenem-non-susceptible isolates indicating that this method lacks specificity. Imipenem and meropenem showed better specificity (23 % and 41 %, respectively) suggesting that all three antibiotics results should be used to enhance specificity for CRE detection. It should be noted however, that in instances where the phenotypic method indicates resistance and the PCR result is negative for all genes tested, resistance may be due to other carbapenemases, AmpC and or ESBL gene/s not screened for by the PCR. Alternatively, other mechanisms of resistance may be present e.g. efflux pumps and porin mutations. The former statement may be supported for example by the low cefoxitin susceptibility rate (10.8 %) suggesting that plasmid-mediated AmpC enzymes are very prevalent in our sample population. Similarly, the low susceptibility rates for the penicillins and cephalosprins (0.6–13 %) are indicative of ESBLs. We have not screened for these. However, the possibility remains that these isolates could in fact be non-CPE. This is worrying as phenotypic methods are routinely utilised and if ertapenem non-susceptibility is strictly used as an indication to treat patients with carbapenems even though a carbapenemase is not present, this would result in further implications towards potential antimicrobial resistance. This stresses the need for specific tests that can routinely be used.

Overall the phenotypic method used for the detection of carbapenemase production was highly sensitive for ertapenem (98 %) but the specificity was low (13 %), an indication that the method may possibly be over-reporting results potentially influencing clinicians to prescribe unnecessary antibiotics. For imipenem and meropenem, the specificities were satisfactory but the sensitivities were not. These antibiotics would therefore not be the ideal antibiotic of choice for this purpose.

## Conclusion

We have shown an increase in carbapenemase-producing Enterobacteriaceae over a three year period with close to 40 % of all isolates received being *bla*_NDM_-positive. Controlling the spread and limiting the impact of CPEs in South Africa will require intensive efforts in both the public and private healthcare sectors. The use of phenotypic methods for detection of CPEs using ertapenem is sensitive but lacks specificity and can be used if there is no access to molecular methods. However, conventional AST methods coupled with molecular genotypic testing for the detection of carbapenemase-producing Enterobacteriaceae is more definitive and therefore advantageous.

## Abbreviations

AmpC: Ampicillin class C (AmpC)
AST: Antimicrobial susceptibility testing
CLSI: Clinical and Laboratory Standards Institute
CPE: Carbapenemase-producing enterobacteriaceae
CRE: Carbapenem-resistant enterobacteriaceae
ESBL: Extended-spectrum beta-lactamases (ESBL)
KPC: *Klebsiella pneumoniae* carbapenemases
MBL: Metallo-beta-lactamases
MIC: Minimal inhibitory concentration
PCR: Polymerase chain reaction

## References

[1] Gupta N (2011). Carbapenem-resistant Enterobacteriaceae: epidemiology and prevention. Clin Infect Dis.

[2] Spellberg B (2008). The epidemic of antibiotic-resistant infections: a call to action for the medical community from the Infectious Diseases Society of America. Clin Infect Dis.

[3] Kocsis B, Szabó D. Antibiotic resistance mechanisms in Enterobacteriaceae. Microbial pathogens and strategies for combating them: science, technology and education. Vol. 1 ISBN: 978-84-939843-9-7. 2013: Formatex Research Center.

[4] Falagas ME, Kasiakou SK (2005). Colistin: the revival of polymyxins for the management of multidrug-resistant gram-negative bacterial infections. Clin Infect Dis.

[5] Falagas ME, Kopterides P (2007). Old antibiotics for infections in critically ill patients. Curr Opin Crit Care.

[6] Falagas ME, et al. Antibiotic treatment of infections due to carbapenem-resistant enterobacteriaceae: systematic evaluation of the available evidence. Antimicrob Agents Chemother. 2014;58(2):654-63. 10.1128/AAC.01222-13 PMC3910850 24080646

[7] Livermore DM. My CRE is a CPO, is yours?, in Public Health England, Antimicrobial Resistance and Healthcare Associated Infections Reference Unit (AMRHAI) News. UK: 2013. PHE publications gateway number: 2013137.

[8] Queenan AM, Bush K (2007). Carbapenemases: the Versatile beta-lactamases. Clin Microbiol Rev.

[9] Livermore DM, Woodford N (2006). The beta-lactamase threat in Enterobacteriaceae, Pseudomonas and Acinetobacter. Trends Microbiol.

[10] Rodriguez-Martinez JM (2011). Plasmid-mediated quinolone resistance: an update. J Infect Chemother.

[11] Rodriguez-Martinez JM (2010). VIM-19, a metallo-beta-lactamase with increased carbapenemase activity from Escherichia coli and Klebsiella pneumoniae. Antimicrob Agents Chemother.

[12] Fernández-Cuenca F (2012). Production of a plasmid-encoded OXA-72 β-lactamase associated with resistance to carbapenems in a clinical isolate Acinetobacter junii. Int J Antimicrob Agents.

[13] Carrer A (2008). Spread of OXA-48-positive carbapenem-resistant Klebsiella pneumoniae isolates in Istanbul, Turkey. Antimicrob Agents Chemother.

[14] Cuzon G, Bonnin RA, Nordmann P (2013). First identification of novel NDM carbapenemase, NDM-7, in Escherichia coli in France. PLoS One.

[15] Gottig S (2013). Detection of NDM-7 in Germany, a new variant of the New Delhi metallo-beta-lactamase with increased carbapenemase activity. J Antimicrob Chemother.

[16] Hayakawa K, et al. Molecular and epidemiological characterization of iMP-type metallo-beta-lactamase-producing enterobacter cloacae in a Large Tertiary Care Hospital in Japan. Antimicrob Agents Chemother. 2014;58(6):3441-50. 10.1128/AAC.02652-13 PMC4068452 24709261

[17] Hornsey M, Phee L, Wareham DW (2011). A novel variant, NDM-5, of the New Delhi metallo-beta-lactamase in a multidrug-resistant Escherichia coli ST648 isolate recovered from a patient in the United Kingdom. Antimicrob Agents Chemother.

[18] Koh TH, Wang GCY, Sng L (2004). IMP-1 and a novel metallo-beta-lactamase, VIM-6, in fluorescent pseudomonads isolated in Singapore. Antimicrob Agents Chemother.

[19] Leung GHY, et al. Persistence of related bla-IMP-4 metallo-betalactamase producing Enterobacteriaceae from clinical and environmental specimens within a burns unit in Australia - a six-year retrospective study. Antimicrob Resist Infect Control. 2013;2(35). doi: 10.1186/2047-2994-2-35. 10.1186/2047-2994-2-35 PMC3878348 24345195

[20] Patzer JA (2009). Emergence and persistence of integron structures harbouring VIM genes in the Children’s Memorial Health Institute, Warsaw, Poland, 1998–2006. J Antimicrob Chemother.

[21] Poirel L (2010). Emergence of metallo-beta-lactamase NDM-1-producing multidrug-resistant Escherichia coli in Australia. Antimicrob Agents Chemother.

[22] Richter SN (2011). Transfer of KPC-2 Carbapenemase from Klebsiella pneumoniae to Escherichia coli in a patient: first case in Europe. J Clin Microbiol.

[23] Robin F (2010). Novel VIM metallo-beta-lactamase variant from clinical isolates of Enterobacteriaceae from Algeria. Antimicrob Agents Chemother.

[24] Shet V (2011). IMP metallo-beta-lactamase-producing clinical isolates of Enterobacter cloacae in the UK. J Antimicrob Chemother.

[25] Yong D (2009). Characterization of a new metallo-beta-lactamase gene, bla(NDM-1), and a novel erythromycin esterase gene carried on a unique genetic structure in Klebsiella pneumoniae sequence type 14 from India. Antimicrob Agents Chemother.

[26] Zarfel G (2011). Carbapenemases in Enterobacteria, Hong Kong, China, 2009. Emerg Infect Dis.

[27] Brink AJ (2011). Emergence of New Delhi metallo-beta-lactamase (NDM-1) and Klebsiella pneumoniae carbapenemase (KPC-2) in South Africa. J Clin Microbiol.

[28] Govind C (2013). NDM-1 imported from India - first reported case in South Africa. SAMJ.

[29] Brink AJ (2013). Emergence of OXA-48 and OXA-181 carbapenemases among Enterobacteriaceae in South Africa and evidence of in vivo selection of colistin resistance as a consequence of selective decontamination of the gastrointestinal tract. J Clin Microbiol.

[30] Lowman W (2011). NDM-1 has arrived: First report of carbapenem resistance mechanism in South Africa. SAMJ.

[31] Jacobson RK (2015). Molecular Characterisation and Epidemiolgical Investigation of an Outbreak of blaOXA-181 carbapenemase-producing isolates of Klebsiella pneumoniae in South Africa. SAMJ.

[32] Singh-Moodley A, Ekermans P, Perovic O (2015). Emerging Carbapenem-Resistant Enterobacter cloacae Producing OXA-48-, VIM- and IMP-Type-β-lactamases in Eastern Cape Hospitals in South Africa. Open J Med Microbiol.

[33] Chea N (2015). Improved Phenotype-Based Definition for Identifying Carbapenemase Producers among Carbapenem-Resistant Enterobacteriaceae. Emerg Infect Dis.

[34] Clinical and Laboratory Standards Institute (CLSI) guidelines - Performance Standards for Antimicrobial Susceptibility Testing. in CLSI document M100. 2016.

[35] Brink A (2012). The speread of carbapenem-resistant Enterobacteriaceae in South Africa: Risk factors for acquisition and prevention. S Afr Med J.

[36] Docquier JD (2003). On functional and structural heterogeneity of VIM-type metallo-beta-lactamases. J Antimicrob Chemother.

[37] Tato M (2010). Carbapenem Heteroresistance in VIM-1-producing Klebsiella pneumoniae isolates belonging to the same clone: consequences for routine susceptibility testing. J Clin Microbiol.

